# Long non-coding RNAs expression profile and functional analysis of acute ischemic stroke

**DOI:** 10.1097/MD.0000000000022964

**Published:** 2020-12-11

**Authors:** Jia Li, Miao Hao, Ben Yang, Tianji Shi, Yingyu Zhang, Jingqi Feng, Jiajun Chen

**Affiliations:** a Department of Neurology; b Scientific Research Center; c Ophthalmology, China-Japan Union Hospital of Jilin University, Changchun, P.R. China.

**Keywords:** acute ischemia stroke, long non-coding RNA, pathway analysis

## Abstract

Long non-coding RNAs (lncRNAs) have been evidenced to be associated with the development of multiple diseases. However, the expression pattern and function of lncRNAs in acute ischemic stroke remain unclear. To determine the differential expression of lncRNAs in acute ischemic stroke, we analyzed the expression profile of lncRNAs by high-throughput sequencing analysis. Gene Ontology (GO) and pathway analyses were employed to analyze the gene function and identify enriched pathways of the differentially expressed lncRNAs. We also built an lncRNA-mRNA expression correlation network and verified the interactions of selected lncRNAs in acute ischemic stroke. To further confirm the results of the expression profile, 6 differentially expressed lncRNAs were randomly selected and quantitative RT-PCR (qRT-PCR) performed. We identified 44,578 aberrantly expressed lncRNAs, including 228 upregulated and 16 downregulated lncRNAs. The qRT-PCR results showed that ENSG00000269900, ENSG00000196559, ENSG00000202198, ENSG00000226482, ENSG00000260539 (up), and XLOC_013994_2 (down) were abnormally expressed, which was consistent with the sequencing results. The upregulated expression of lncRNA ENSG00000226482 may activate the adipocytokine signaling pathway, resulting in acute ischemia stroke. In summary, we analyzed the lncRNAs expression profile in acute ischemic stroke patients and identified the functions and enriched metabolic pathways, proposing new insights into the diagnostic and therapeutic biomarkers for this disease.

## 1 Introduction

Acute ischemic stroke accounts for approximately 80% of all strokes and is characterized by high levels of morbidity, morbidity, mortality, and recurrence rate, and poor prognosis.^[1]^ To improve the quality of life of patients, it is urgent to identify new biomarkers for early diagnosis and treatment of acute ischemia stroke and to clarify its potential molecular mechanism. However, most of the current clinical studies on the pathogenesis of acute ischemia stroke remain at the level of imaging or pathology. With the development of sequencing technology, biomolecules such as genes and non-coding RNAs have been suggested to play crucial roles in the process of acute ischemia stroke.

Long noncoding RNAs (lncRNAs) are a group of RNA transcripts >200 nucleotides (nt) without coding potential. Many recent studies evidenced that lncRNAs has vital functions in a number of biological processes, such as cell metabolism, immune response, tumorigenesis, and development.^[2]^ LncRNAs regulate gene expression by interacting with DNA, RNA, or protein molecules, ultimately affecting cell life activities.^[3,4]^ To date, the studies on lncRNA have been focused on tumors and neurodegenerative diseases,^[5,6]^ and lncRNAs have been reported to be potentially involved in the process of ischemic stroke injury, leading to their abnormal post-ischemic expression.^[7,8]^ However, the involvement of the expression of lncRNAs in stroke is unclear, and the underlying pathogenesis and molecular mechanisms of acute ischemic stroke are not well understood.^[9–12]^ Therefore, in the present study, we aimed to identify dysregulated lncRNAs in acute ischemic stroke patients and clarify their potential functions. Our data revealed that the aberrant expression of lncRNAs may contribute to the incidence and development of acute ischemic stroke. Moreover, we suggested new molecular markers and therapeutic targets for this disease.

## 2 Methods

### 2.1 Patients’ enrollment and sample collection

The study was approved by the Ethics Committee of the China-Japan Union Hospital of Jilin University, China (No. 2018122503). All patients enrolled in this study provided informed consent for publication of the case. Eighty participants were included in this study, consisting of a test group of outpatients and inpatients from the Neurology Department of the aforementioned hospital from July to September 2017. A control group of subjects was recruited from the Neurology Department or the Physical Examination Department.

The patients were diagnosed as acute cerebral infarction aged 55 to 70 years, with an onset of stroke <1 week, with no other systemic diseases except hypertension and diabetes. Patients presented with clear symptoms of neurological deficit. magnetic resonance imaging or computed tomography confirmed the diagnosis of ischemic stroke. The individuals in the control group had no diseases. Ethylenediaminetetraacetic acid (EDTA) anticoagulated vacuum blood collection tubes were used to collect blood (3 mL) from the cubital veins, followed by extraction of total RNA.

### 2.2 Total RNA extraction

Total RNA was extracted using TRIzol reagent according to manufacturer's protocol. The quantity and quality of RNA were measured by NanoDrop.

### 2.3 RNA library construction and lncRNA sequencing

Ribo-Zero rRNA Removal Kits (New England Biolabs, lnc., Massachusetts) and TruSeq Stranded Total RNA Library Prep Kit (Illumina) were used to prepare and construct RNA libraries. Then, the libraries were controlled and quantified by the BioAnalyzer 2100 System (Agilent Technologies). Next, 10 pM of the libraries was denatured into single-stranded DNA molecules, which were captured on Illumina flow cells, expanded in situ as clusters, and finally sequenced on Illumina HiSeq Sequencer (150 cycles) according to the manufacturer's instructions.

### 2.4 LncRNA sequencing analysis

Paired-end reads were harvested from Illumina HiSeq 4000 sequencer after quality filtering. First, Q30 was used to perform quality control. Then, lncRNAs were discovered and distinguished by Cutadapt software (v1.9.2), bowtie software (v2.2.4), find_lnc software (v1.2), and STAR software (v2.5.1b) (SPSS 17.0; SPSS Inc., Chicago, IL).^[13–16]^ Raw junction reads for all samples were normalized by total reads number and log2-transformed. Differential expressed lncRNAs were screened according to the threshold values of |fold change| ≥2, and *P* < .05. Using Heatmap2 in R software, we performed clustering analysis of the differentially expressed lncRNAs with standardized reads to visualize the differential expression of lncRNAs in the samples of the 2 groups. Volcano plot of differentially expressed lncRNAs (|fold change| ≥2.0 and *P*-value ≤ .05.) were drawn. GO enrichment analysis (http://www.geneontology.org) and Kyoto Encyclopedia of Genes and Genomes (KEGG) pathway enrichment analysis (https://www.kegg.jp) were used to annotate the functions of the targets genes.^[17]^

### 2.5 Quantitative real-time RT-PCR

Total RNA was extracted using TRIzol reagent. mRNA was reverse-transcribed by a PrimeScript RT Reagent Kit (TaKaRa, Japan) following the manufacturer's instruction. Then, qPCR was performed by a SYBR Green qPCR kit (Roche, USA).^[18]^ Specific primers of each gene are listed in Table 1. Relative mRNA expression was normalized to housekeeping gene GAPDH, and data were calculated by the Livak method (2^–ΔΔCt^).^[19]^ All analyses of the samples were conducted in triplicate.

**Table 1:** Primers used for qRT-PCR.

lncRNA	Forward primer (5′-3′)	Reverse primer (5′-3′)
ENSG00000269900	AAGTCCGCCAAGAAGCGTATCC	GCACTGCCTGCGTAACTAGAGG
ENSG00000196559	AAGGAGCAAGCGTTCCGACAAG	CTGACTGAGGAGCACTGACAACAC
ENSG00000202198	GGCGATCTGGCTGCGACATC	GACGCACATGGAGCGGTGAG
ENSG00000226482	CTAGGCCAGGAGTTCGAGACCAG	TGACACGACCTCGGCTCACTG
ENSG00000260539	TGGTCCTTCCTCCACAGACTTCAG	TCACCACCAGCCTGAGCAGAC
XLOC_013994_2	GGCCGTGCGTACTTAGACATGC	GTCCGTCCGTCGTCCTCCTC
XLOC_010820_2	GCCGAACTCAGTGCGGACAC	GCTGTAGTGCGCTATGCCGATC

lncRNA = long non-coding RNAs; qRT-PCR = quantitative RT-PCR.

### 2.6 Statistical analysis

All statistical analyses were performed by SPSS software (version 17.0). The results were expressed as mean ± standard deviation (SD). Student's *t* test was employed as appropriate to analyze the expression levels between 2 groups, *P* < .05 was considered to indicate a statistically significant difference.

## 3 Results

### 3.1 LncRNAs expression profile of acute ischemic stroke

**Figure 1:**
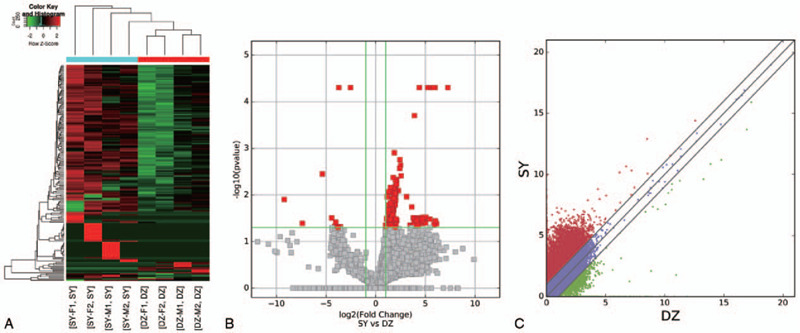
Differences in lncRNAs expression profiles between the acute ischemia stroke and sham groups. (A) Hierarchical cluster analysis of 44,578 differentially expressed lncRNAs. Each column represents one sample; each row represents one differentially expressed lncRNAs. Red strips represents up-regulated high relative expressions, green strips represents down-regulated expressions, and the deeper the color, the high the significant difference. The dendrogram on top shows the clustering of the samples. The dendrogram on the left shows the clustering of the genes. (B) Volcano plots show the distributions of lncRNAs between the acute ischemia stroke and sham groups. The horizontal line corresponds to a 5-fold (log5 scaled) change up or down, and the vertical line represents a *P*-value of <.05 (−log10 scaled). The red points on the plot represent the differentially expressed lncRNAs with a 5-fold change up or down in expressed lncRNAs with statistical significance (*P* < .05). (C) The scatter plot shows the differences in lncRNAs expression between the acute ischemia stroke and sham groups. The values plotted on the *x* and *y* axes are the averaged normalized signal values between the acute ischemia stroke and sham groups (log2 scaled). The gray lines represent fold change. The lncRNAs above the top gray line and below the bottom gray line indicate a >2 fold change of lncRNAs between the 2 compared samples. lncRNAs = long non-coding RNAs.

To observe the differential expression of lncRNAs in patients with acute ischemic stroke, 40 participants including 20 patients and 20 normal controls were used for hierarchical clustering analysis. The experimental group (10 men and 10 women) was divided into 4 groups by sex, and the normal group (10 men and 10 women) was divided into 4 groups by sex. As shown in Fig. 1, we detected a total of 44,578 lncRNAs, including 228 upregulated lncRNAs and 16 downregulated lncRNAs (|fold change| ≥2, *P* < .05). The differentially expressed lncRNAs perfectly distinguished the group with the acute ischemic stroke (SY) from the control group (DZ) (Fig. 1).

The top 20 dysregulated lncRNAs are summarized in Table 2. Out of the group of lncRNAs that were downregulated, lncRNA XLOC_010820_2 and XLOC_013994_2 showed the greatest degree of demonstrated downregulation, with 911573.8- and 840.8-fold decrease, respectively. Of those that were upregulated, lncRNA ENSG00000243039 and ENSG00000202198 had the highest degree of upregulation, 137.0- and 21.1-fold increases, respectively.

**Table 2:** Top 20 differentially expressed lncRNAs between patients with cerebral infarction and control groups.

lncRNA	Source	Fold change	*P*-value	Regulation	Accession gene	Chr
XLOC_010820_2	TCONS	911573.8414	.00355	Down	NEMF	Chr14
XLOC_013994_2	TCONS	840.794837	.01255	Down	N/A	Chr21
ENSG00000243039	Ensembl	136.9513806	.03615	Up	N/A	Chr2
ENSG00000202198	Ensembl	21.08989489	.00005	Up	GSTA4	Chr6
ENSG00000269900	Ensembl	15.14542335	.0002	Up	CCDC107	Chr9
ENSG00000263934	Ensembl	8.732773973	.01095	Up	N/A	Chr17
ENSG00000267790	Ensembl	7.053356695	.00175	Up	N/A	Chr17
ENSG00000230971	Ensembl	7.011339043	.0023	Up	N/A	Chr17
DDX11L11	UCSC_knowngene	6.449467279	.0039	Up	N/A	Chr12
ENSG00000271862	Ensembl	6.411402579	.0027	Up	N/A	Chr5
ENSG00000234423	Ensembl	5.990930097	.00465	Up	N/A	Chr2
ENSG00000236908	Ensembl	5.685195655	.00805	Up	N/A	Chr12
ENSG00000272046	Ensembl	5.46128331	.04235	Up	N/A	Chr13
ENSG00000267325	Ensembl	5.44325642	.0345	Up	N/A	Chr18
ENSG00000261087	Ensembl	5.354047729	.0059	Up	N/A	Chr8
ENSG00000247699	Ensembl	5.35360241	.0355	Up	FABP6	Chr5
XLOC_011789	TCONS	5.298926073	.03275	Up	N/A	Chr16
LOC101926897	RefSeq	5.259734583	.00485	Up	N/A	Chr13
ENSG00000223486	Ensembl	5.162285744	.00775	Up	N/A	ChrX
ENSG00000226567	Ensembl	5.040371251	.00545	Up	N/A	Chr3

Chr = chromosome; lncRNA = long non-coding RNAs; N/A = not annotated.

### 3.2 GO analyses of lncRNAs

**Figure 2:**
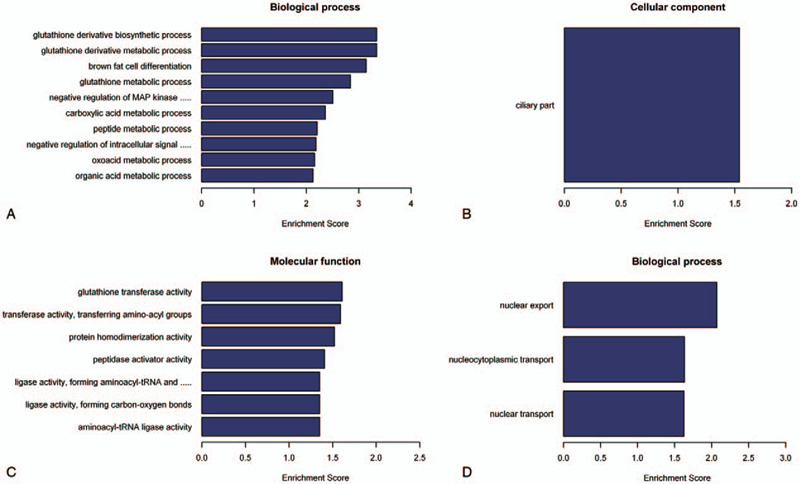
GO analysis of differentially expressed lncRNAs in acute ischemia stroke. The bar plot represented the top 10 Enrichment Score value of the significant enrichment terms. The GO annotations for biological process (A), cellular components (B), and molecular functions (C) of host genes of up-regulated lncRNAs. The GO annotations for biological process (D) of host genes of down-regulated lncRNAs. GO = Gene Ontology; lncRNAs = long non-coding RNAs.

To observe the function of the differential expression of lncRNAs, we used Gene Ontology (GO) annotation. The results revealed that the differentially expressed lncRNAs were enriched in the biological process (BP), molecular function (MF), and cellular component (CC). As can be seen in Fig. 2, 184 items were obtained in patients with acute ischemic stroke. Among them, upregulated lncRNAs involved 173 biological processes, 7 molecular functions, and only 1 cellular component. The downregulated expression of lncRNAs included 3 items in the biological process category, but no molecular function and cellular component were involved. Furthermore, 4 functions with the most enriched lncRNAs were glutathione derivative biosynthetic process (GO: 1901685; Ontology: biological process; *P* = .000453994) (Fig. 2A upregulated lncRNAs), ciliary part (GO: 0044441; Ontology: molecular function; *P* = .02885975) (Fig. 2B upregulated lncRNAs), glutathione transferase activity (GO: 0004364; Ontology: cellular component; *P* = .02461007) (Fig. 2C upregulated lncRNAs), and nuclear export (GO: 0051168; Ontology: biological process; *P* = .008499287) (Fig. 2D downregulated lncRNAs).

### 3.3 KEGG pathway enrichment of lncRNAs

**Figure 3:**
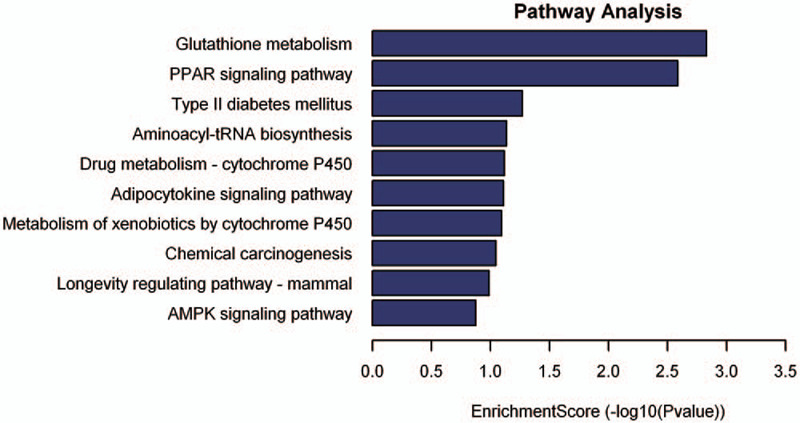
KEGG pathway analysis of up-regulated lncRNAs. Top 10 pathways of up-regulated lncRNAs in acute ischemia stroke. The vertical axis represents the pathway category and the horizontal axis represents the enrichment score (−lg(*P* value)) of the pathway. LgP was the logarithm of *P*-value, and *P* < .05 was considered significant. KEGG = Kyoto Encyclopedia of Genes and Genomes, lncRNAs = long non-coding RNAs.

To explore the role of dysregulated lncRNAs in acute ischemic stroke-related lncRNAs regulation and metabolic pathways, we performed KEGG pathway analysis. As can be observed in Fig. 3, aberrantly expressed lncRNA were involved in upregulated pathways, including glutathione metabolism, peroxisome proliferator-activated receptor (PPAR) signaling pathway, Type II diabetes mellitus, aminoacyl-tRNA biosynthesis, drug metabolism-cytochrome P450, adipocytokine signaling pathway, metabolism of xenobiotics by cytochrome P450, chemical carcinogenesis, the longevity regulating pathway-mammal, and the AMPK signaling pathway (*P* < .05). Whereas downregulated expression of lncRNAs have no enriched pathways.

**Figure 4:**
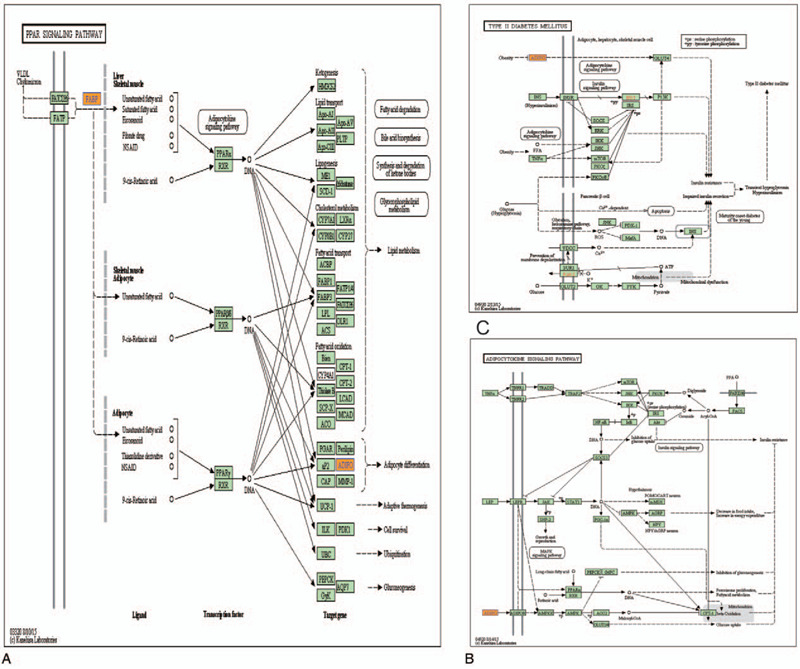
Mapping of pathways of lncRNAs expression pattern involved in PPAR signaling pathway (A), Type II diabetes mellitus (B), and Adipocytokine signaling pathway (C). Yellow marked nodes are associated with down enriched genes, orange marked nodes are associated with up-regulated enriched genes, green nodes have no significance. lncRNAs = long non-coding RNAs; PPAR = peroxisome proliferator-activated receptor.

KEGG analysis identified 228 differentially upregulated lncRNAs in patients with acute cerebral infarction associated with 15 signal pathways (data not shown). Among these pathways, the PPAR signaling pathway (Fig. 4A), Type II diabetes mellitus (Fig. 4B), and the adipocytokine signaling pathway (Fig. 4C) were 3/15 pathways obtained by the KEGG analysis. All of them were involved in the regulation of the expression of ADIPO, which is an important factor in the pathogenesis of acute cerebral infarction.

### 3.4 qRT-PCR results validation

**Figure 5:**
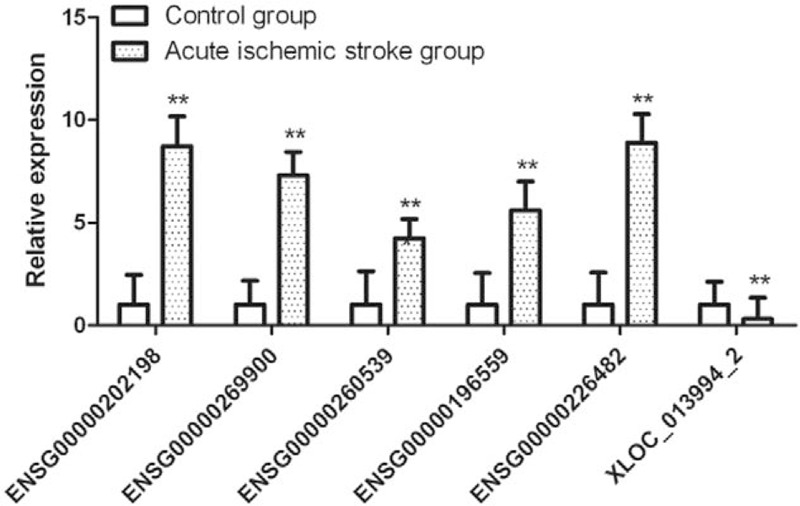
qRT-PCR validation of 6 candidate lncRNAs. The relative expression level of 6 lncRNAs including ENSG00000269900, ENSG00000196559, ENSG00000202198, ENSG00000226482, ENSG00000260539, and XLOC_013994_2, which was determined using qRT-PCR between the acute ischemia stroke group (s = 30) and control group (n = 15). Data are presented as mean ± standard deviation (SD). ∗*P* < .05, ∗∗*P* < .01, versus control group. lncRNAs = long non-coding RNAs; qRT-PCR = quantitative RT-PCR.

To confirm the reliability of the microaary data, we enlarged the sample sizes and selected 6 differentially expressed lncRNAs (5 upregulated and 1 downregulated) and measured their expression level using RT-qPCR among 30 healthy controls and 30 patients including those 10 normal controls and 10 patients in the microarry. As illustrated in Fig. 5, ENSG00000269900, ENSG00000196559, ENSG00000202198, ENSG00000226482, and ENSG00000260539 were significantly upregulated, whereas XLOC_013994_2 was significantly downregulated in patients with acute cerebral infarction. The results of quantitative RT-PCR (qRT-PCR) were consistent with those of the expression profile. All 6 lncRNAs were differentially expressed with the same trend (up- or downregulated).

## 4 Discussion

LncRNAs, a group of RNA transcripts >200 nt, lack an open reading frame. Although lncRNAs do not encode proteins, they can regulate their epigenetic, transcriptional, and post-transcriptional levels. Currently, lncRNAs have been shown to be critically involved in many diseases, such as tumors, nervous system diseases, cardiovascular diseases, and mental disorders. However, the specificities in the lncRNAs expression in patients with acute cerebral infarction are still unclear. To investigate their potential biological functions, we performed high-throughput sequencing and GO and KEGG pathways analyses. The results showed that a larger amount of differentially expressed lncRNAs were present in the patients with acute ischemic stroke, compared with control groups. We detected a total number of 228 upregulated and 16 downregulated lncRNAs with significant abnormal expression. As shown in supplementary data, GSTA4, associated with ENSG00000202198, was enriched mainly in the metabolism and biosynthesis of glutathione derivative (GO: 1901685) (GO: 1901687). ADIPOQ, associated with ENSG00000226482, was enriched mainly in the negative regulation of gluconeogenesis (GO: 0045721), the cellular response to epinephrine stimulus (GO: 0071872), the regulation of protein kinase A signaling (GO: 0010738), the regulation of cAMP-dependent protein kinase activity (GO: 2000479), and the low-density lipoprotein receptor particle metabolic process (GO: 0032799). GLG1, associated with ENSG00000260539, was predominantly enriched in the regulation of chondrocyte differentiation (GO: 0032330). NEMF, associated with XLOC_010820_2, was enriched mostly in the nuclear, nucleocytoplasmic, and nuclear transport (GO: 0051168) (GO: 0006913) (GO: 0051169).

KEGG pathway analysis combines not only genes, chemicals, and various network informations at the genetic levels, but also conducts pathway analysis of differentially expressed lncRNAs involved in acute cerebral infarction. Among them, GSTA4, associated with ENSG00000202198, in the glutathione metabolism (PathwayID: hsa00480), drug metabolism—cytochrome P450 (PathwayID: hsa00982), metabolism of xenobiotics by cytochrome P450 (PathwayID: hsa00980), and chemical carcinogenesis (PathwayID: hsa05204). ADIPOQ, associated with ENSG00000226482, is involved in the PPAR signaling pathway (PathwayID: hsa03320), type II diabetes mellitus (PathwayID: hsa04930), the adipocytokine signaling pathway (PathwayID: hsa04920), the longevity regulating pathway—mammal, the AMPK signaling pathway, and non-alcoholic fatty liver disease (NAFLD) (PathwayID: hsa04932). GLG1, associated with ENSG00000260539, plays a role in cell adhesion molecules (CAMs) (PathwayID: hsa04514).

Based on the findings of the GO annotation and KEGG pathways analysis, we focused our attention on ENSG00000226482, which may regulate ADIPOQ by expression upregulation, resulting in a variety of pathways, such as adipocytokine signaling pathways, and is associated with acute cerebral infarction.

ADIPOQ (adiponectin, or C1Q and collagen domain containing, GBP-28, apM1, AdipoQ, etc), is maintained at a concentration of 3 to 30 μg/mL in plasma and mainly secreted by mature adipocytes. Adiponectin has many important physiological functions, including regulate energy metabolism, increase in insulin sensitivity, promotion of its production, and even effects on atherosclerosis and angiogenesis. For example, adiponectin inhibited NF-κB and its transcription factors expression, protecting the aorta from atherosclerotic injury by reducing inflammation.^[20]^ Adiponectin accumulated in the vasculature by T-cadherin, protecting neointima proliferation, and atherosclerosis.^[21]^ Adiponectin prevented atherosclerosis by reducing oxidative stress.^[22]^

Adiponectin can also exert physiological effects in combination with adiponectin receptor 1 (AdipoR1) and adiponectin receptor 2 (AdipoR2). For example, adiponectin attenuated neuronal apoptosis through AdipoR1/APPL1/LKB1/AMPK signaling pathway.^[23]^ Adiponectin aggravated atherosclerosis by inhibiting the AdipoR1-AMPK-iNOS pathway in adventitial fibroblasts.^[24]^ Furthermore, PPAR caused an improvement in obesity-induced insulin resistance by activating adiponectin-AdipoRs.^[25]^ These data are consistent with the sequencing results of our study. We found that adiponectin is involved in the adipocytokine signaling primarily through activation of the adenosine kinase (AMPK) and PPAR pathways.

Adiponectin regulates blood glucose homeostasis by AdipoRs stimulation. In the body's lipid metabolism, adiponectin is positively correlated with high-density lipoprotein but negatively correlated with triglycerides and low-density lipoprotein.^[26,27]^ In addition, adiponectin is critically involved in ischemic stroke.^[28,29]^ Pera et al^[30]^ found that the plasma adiponectin levels were significantly reduced in patients with ischemic stroke, and the decrease of adiponectin levels was a dynamic process. Therefore, based on the results of our study, we further speculated that the adiponectin synthesis may increase after stroke, and the pathological process can lead to the consumption of adiponectin. The adiponectin level in the acute phase decreases dynamically, indicating an increase in its consumption. These results indicated that the level of adiponectin in patients could be used as an important marker for whether patients have acute stroke. However, it is difficult to accurately detect it because the level of adiponectin depends on the 2 factors of adiponectin synthesis and consumption. Therefore, the expression of ENSG00000202198, upstream regulator of adiponectin, might provide promising to the early diagnosis of acute stroke.

Clinical studies have shown that plasma adiponectin levels <4 μg/mL are independent risk factors for increased mortality in stroke patients. Due to the low anti-inflammatory capacity, patients with low plasma adiponectin have an increased risk of mortality within 5 years after the first ischemic stroke. Carbone et al^[31]^ established that serum leptin and leptin/adiponectin ratios increased on day 1 after the acute ischemic stroke and were inversely correlated with the radiological and clinical parameters at all follow-up time points. The results of this study suggested that the leptin/adiponectin ratio could predict the outcomes in patients with atherothrombotic acute ischemic stroke and served as a prognostic biomarker. However, since the adiponectin monomer is present only in adipocytes, the measurement of peripheral blood multimeric adiponectin alone does not reflect its exact level. Our results showed that the expression of ENSG00000202198 is significantly up-regulated, which is related to adiponectin, indicating that ENSG00000202198 expression might reflect ADIPOQ level in patients with ischemic stroke. Therefore, ENSG00000226482 is expected to become a potential biomarker and target for the diagnosis and treatment of cerebral infarction. On the one hand, more groups should be involved, including normal group, arteriosclerosis patient group, acute cerebral infarction patient group, and chronic cerebral infarction patient group, and each group is divided into high adiponectin group/low adiponectin group. On the other hand, qPCR should be validated with more samples. Then, in-depth investigations should be conducted on the function and mechanism of ENSG00000226482 in future.

In summary, we obtained the expression profile of lncRNAs in patients with acute cerebral infarction by high-throughput sequencing and identified a series of significantly differentially expressed lncRNAs. Then, we evaluated the potential functions of these lncRNAs by GO annotation and KEGG pathways analysis. The data suggested that ENSG00000226482 may upregulate the ADIPOQ expression, active multiple signaling pathways, abnormal lipid metabolism, diabetes, and atherosclerosis, leading to acute ischemic stroke. Therefore, ENSG00000226482 can serve as a potential diagnostic biomarker or drug treatment target for acute ischemic stroke.

## Author contributions

**Data curation:** Jia Li, Ben Yang, Tianji Shi.

**Design:** Jiajun Chen.

**Formal analysis:** Miao Hao, Ben Yang, Tianji Shi.

**Investigation:** Jingqi Feng, Yingyu, Zhang.

**Writing – original draft:** Jia Li, Jiajun Chen.

**Writing – review & editing:** Miao Hao.
